# Long-Term Weight Variability Is Associated with Higher Triglyceride Concentrations After Accounting for Long-Term Weight Slope

**DOI:** 10.3390/nu18162605

**Published:** 2026-08-10

**Authors:** Miaomiao Xu, Huiguo Wang, Lin Zhu, Xiaoguang Liu

**Affiliations:** 1 College of Physical Education, Guangdong University of Education, Guangzhou 510303, China; miaomiaoxu@gzucm.edu.cn; 2 College of Sports and Health, Guangzhou Sport University, Guangzhou 510500, China; 3 Research Center for Innovative Development of Sports and Healthcare Integration, Guangzhou Sport University, Guangzhou 510500, China

**Keywords:** weight variability, triglycerides, longitudinal analysis, lipid metabolism

## Abstract

**Background**: Long-term fluctuations in body weight may reflect changes in energy balance, illness, or intentional weight loss. However, their association with subsequent triglyceride concentrations remains uncertain. **Objective**: To examine the association between long-term weight variability and subsequent triglyceride concentrations while accounting for directional weight change. We also assessed the robustness of the findings to adjustment for adiposity, missing data, and socioeconomic and lifestyle covariates. **Methods**: We analyzed adults from the China Health and Nutrition Survey who had at least three measured weights during 1989–2006 and a triglyceride measurement in 2009. The core complete-case analysis included 5191 participants and adjusted for baseline age, sex, weight slope, number of weight measurements, and working status in 2009. Weight variability was assessed using the coefficient of variation (CV; per 1 SD increment, standardized among 9500 eligible participants) and average successive variability (ASV; per 5 kg increment). **Results:** In the core model, each 1 SD increment in CV was associated with 2.7% higher triglyceride concentrations (β = 0.026, 95% CI: 0.007 to 0.045; *p* = 0.007). Each 5 kg increment in ASV was associated with 10.7% higher triglyceride concentrations (β = 0.102, 95% CI: 0.057 to 0.147; *p* < 0.001). After further adjustment for baseline BMI in the same BMI-complete sample, the corresponding estimates were 3.6% (95% CI: 1.7% to 5.6%) and 5.6% (95% CI: 1.0% to 10.3%), respectively. Multiple imputation yielded similar estimates. A four-knot restricted cubic spline analysis supported an overall association between CV and triglyceride concentrations (*p* = 0.016), with no strong evidence of nonlinearity (*p* = 0.101). Marginal standardization yielded adjusted differences in arithmetic mean triglyceride concentrations of 0.048 mmol/L per 1 SD increment in CV and 0.200 mmol/L per 5 kg increment in ASV. **Conclusions**: Greater long-term weight variability was associated with modestly higher subsequent triglyceride concentrations. Because of the observational design, the heterogeneous causes of weight change, and the possibility of residual confounding, the findings should not be interpreted as causal or independent of obesity.

## 1. Introduction

Obesity and cardiometabolic disorders remain major global health challenges [1]. BMI is widely used to describe body size, whereas repeated body-weight measurements provide complementary information by capturing how an individual’s weight changes over time [2]. It remains uncertain whether long-term within-person weight variability is associated with subsequent lipid concentrations after accounting for the overall direction of weight change.

Weight variability refers to the dispersion of repeated body-weight measurements within an individual [3,4]. It should not be interpreted as synonymous with intentional weight cycling. Similar levels of numerical variability may result from planned weight loss and regain, illness-related changes, pregnancy, differences in measurement timing, or natural long-term fluctuations [5]. These causes may have different metabolic consequences, but they cannot be fully distinguished in most observational cohorts.

Greater weight variability has been associated with cardiovascular events and mortality [6,7,8,9,10]. Evidence regarding intermediate lipid phenotypes is more limited. Any observed association may also reflect differences in underlying weight trajectories, adiposity, health behaviors, or socioeconomic conditions [11,12]. Triglycerides were selected as the prespecified outcome because they are responsive to changes in energy balance and lipid metabolism. They may therefore be particularly sensitive to repeated changes in body weight.

We used data from the China Health and Nutrition Survey (CHNS), which includes repeated bodyweight measurements over an extended period and triglyceride measurements collected in 2009 [13]. We quantified weight variability during 1989–2006 and statistically separated it from each participant’s long-term weight slope. We also evaluated whether the findings were robust to missing data, adiposity, socioeconomic factors, and available lifestyle covariates.

We hypothesized that greater long-term weight variability would be associated with higher subsequent triglyceride concentrations after adjustment for directional weight change. Because the causes of weight variability were not directly observed, the analysis was designed to estimate an association rather than a causal effect.

## 2. Materials and Methods

### 2.1. Study Population and Participant Selection

Data were obtained from the China Health and Nutrition Survey (CHNS), an ongoing longitudinal cohort conducted across multiple provinces in China. Repeated anthropometric measurements collected during 1989–2006 were linked to biomarker data obtained in 2009. Participants were eligible if they were aged ≥18 years at their first included survey wave and had at least three measured weights during the exposure period. A total of 9500 participants met these eligibility criteria. This study was reported in accordance with the relevant STROBE recommendations for observational studies.

Of the 9500 eligible participants, 4297 did not have a triglyceride measurement in 2009. This left 5203 participants with observed outcome data. Twelve of these participants had missing data on working status in 2009. The complete-case sample for the core Model B analysis therefore included 5191 participants.

**Figure 1:**
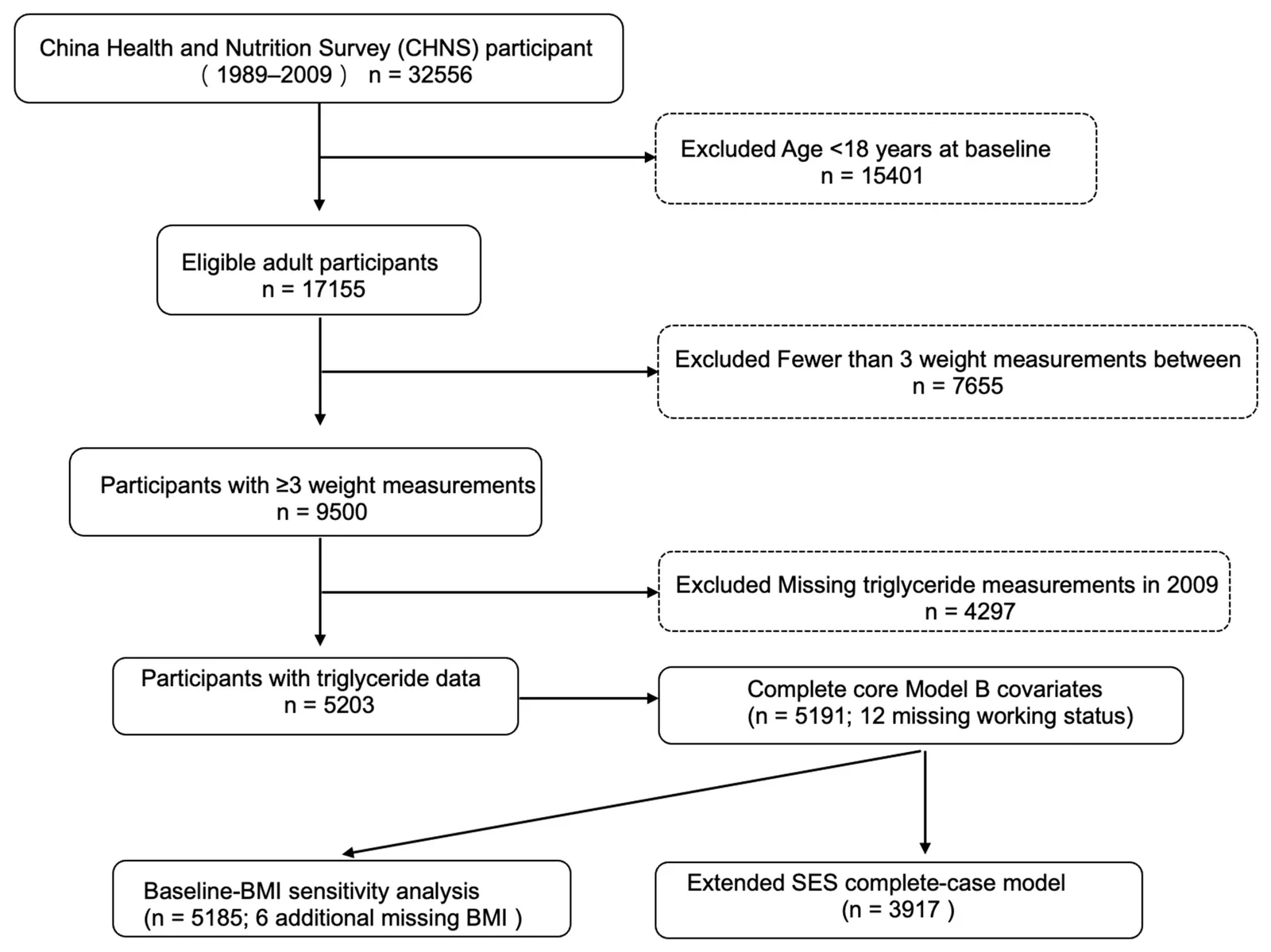
Participant flow and analysis samples in the China Health and Nutrition Survey, 1989–2009.

Two sensitivity analyses used different analytic samples. The baseline-BMI sensitivity analysis included 5185 participants after the exclusion of six additional participants with missing baseline BMI. The extended socioeconomic model additionally included education and individual-income quintile and was based on 3917 complete cases. The multiple-imputation analysis retained all 5203 participants with observed triglyceride measurements (Figure 1).

Because the analytic sample differed across models, the number of participants is reported for each estimate. No participant was excluded on the basis of the magnitude of either weight variability measure.

### 2.2. Assessment of Weight Variability

Body weight was measured at multiple CHNS survey waves between 1989 and 2006 using standardized protocols. All measurements available during this exposure period were ordered by survey year. The number and timing of measurements differed across participants.

For each participant, the coefficient of variation (CV) was calculated as the SD of repeated body-weight measurements divided by the mean body weight and multiplied by 100. For the regression analyses, CV was standardized using the mean and SD among all 9500 eligible participants. The mean CV was 5.850%, and the SD was 3.386 percentage points. CV was modeled per 1 SD increment.

Average successive variability (ASV) was calculated as the mean absolute difference, in kilograms, between consecutive weight measurements ordered by survey year. ASV was modeled per 5 kg increment. Directional weight change was summarized using a participant-specific slope estimated from a linear regression of measured body weight on survey year. The number of weight measurements was included as a covariate to account for differences in measurement opportunity.

### 2.3. Assessment of Triglycerides

Fasting blood samples were collected in 2009 according to standardized CHNS procedures. Serum triglyceride concentrations were measured using the glycerol-phosphate oxidase–peroxidase (GPO-PAP) method on a Hitachi 7600 automated analyzer (Hitachi, Ltd., Tokyo, Japan). Triglyceride concentrations were expressed in mmol/L and were the sole outcome of this study.

Triglycerides were natural log-transformed for regression because of right skew. Coefficients are reported on the log scale and as percent differences, calculated as [exp(β) − 1] × 100. Absolute effects on the mmol/L scale were obtained by sample-average marginal standardization with Duan’s smearing retransformation.

### 2.4. Covariates

Core Model B included baseline age, sex, participant-specific weight slope, number of weight measurements, and working status in 2009. Baseline age and sex were selected because of their established associations with body weight and triglyceride concentrations. Weight slope was included to distinguish within-person weight variability from directional long-term weight change, and the number of weight measurements was included to account for differences in the opportunity to observe weight fluctuations. Working status in 2009 was included as an available concurrent indicator of employment-related social and occupational circumstances, rather than as a direct measure of physical activity. Because working status was measured after the period used to calculate weight variability and concurrently with triglyceride concentrations, it was not treated as a temporally established baseline confounder; its inclusion in Model B should therefore be interpreted as concurrent covariate adjustment rather than baseline confounding control.

Baseline BMI, measured at the first included weight-assessment wave, was added to Model B in a same-sample sensitivity analysis. Highest education level and individual-income quintile, both measured in 2009, were added in an extended socioeconomic sensitivity model. Current smoking, past-year alcohol use, and occupational physical activity, also measured in 2009, were examined in exploratory sensitivity analyses. These 2009 variables were treated as concurrent sensitivity covariates rather than temporally established baseline confounders.

A further contemporaneous dietary sensitivity analysis used individual 3-day average dietary intake data collected in the 2009 CHNS survey, including total energy, carbohydrate, fat, and protein intake. Because dietary intake and triglycerides were measured concurrently in 2009, these variables were not considered temporally established baseline confounders. Within the extended socioeconomic model, we fitted an energy-only model and a primary dietary model additionally adjusted for fat energy percentage. Carbohydrate energy percentage was examined in a separate alternative model because fat and carbohydrate energy percentages were strongly inversely correlated. These analyses were restricted to participants with complete dietary data, with an otherwise identical same-sample reference model.

### 2.5. Statistical Analysis

Separate ordinary least-squares regression models were fitted for CV and ASV, with natural log-transformed triglyceride concentrations as the outcome. CV was modeled per 1 SD increment, and ASV was modeled per 5 kg increment. HC3 heteroskedasticity-consistent standard errors were used for primary inference. Results are reported as β coefficients, 95% confidence intervals (CIs), percentage differences, and two-sided *p* values.

The core Model B served as the primary analysis. For the baseline-BMI sensitivity analysis, Model B was first refitted in the BMI-complete sample and then extended by adding baseline BMI. Relative attenuation of the coefficient was calculated as (βreference − βBMI-adjusted)/βreference × 100. The extended socioeconomic model additionally included education and income. Standard errors clustered at the community level were examined in a separate sensitivity analysis.

**Figure 2:**
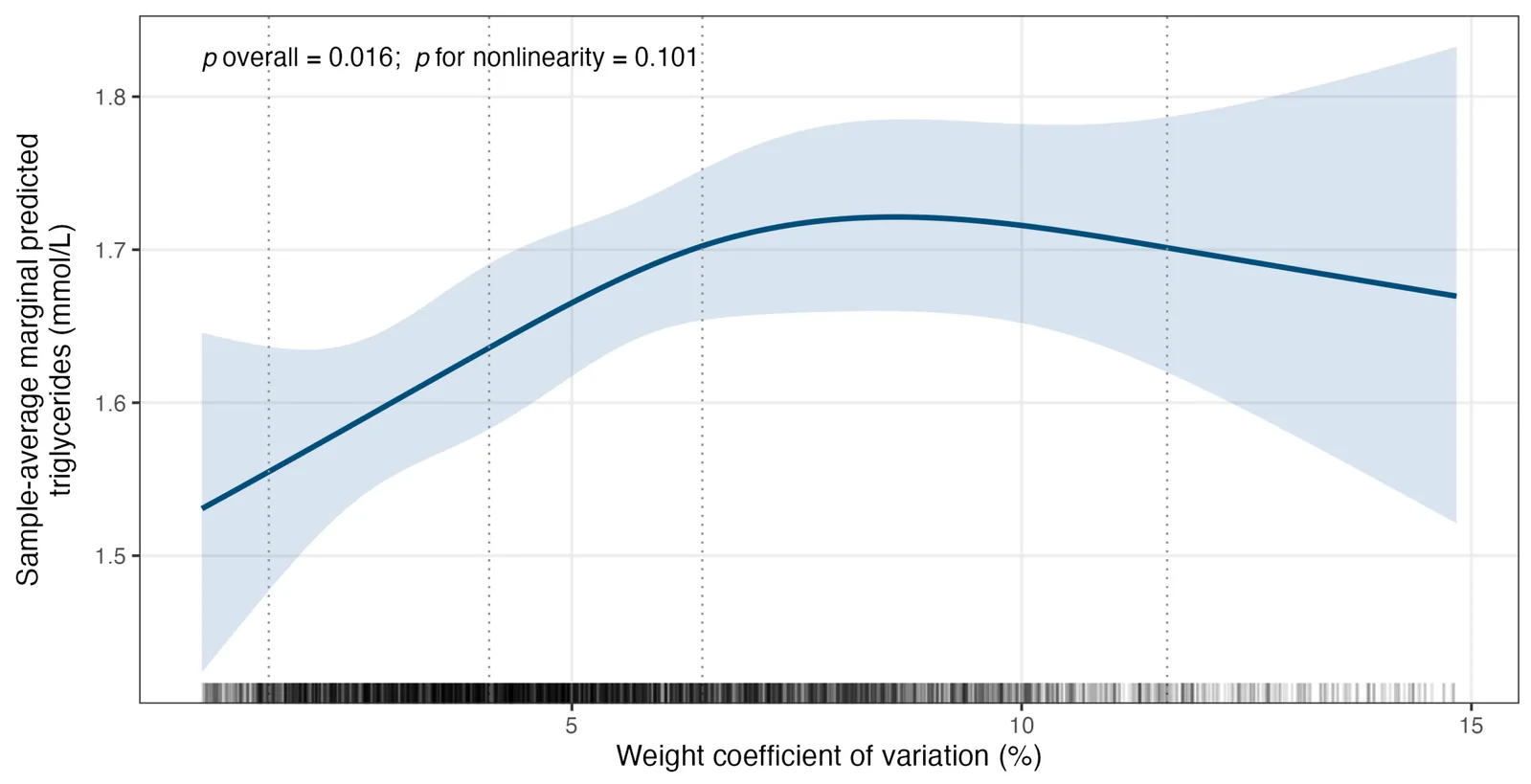
Sample-average marginal predictions of triglyceride concentrations across values of weight coefficient of variation (CV). Predictions were obtained from a restricted cubic spline model fitted to the extended socioeconomic complete-case sample. Predicted values were retransformed to the arithmetic scale (mmol/L) using Duan’s smearing method and then averaged across the sample. The solid line shows the marginal prediction, and the shaded band shows the pointwise 95% confidence interval. The vertical dotted lines mark the four spline knots. Rug marks show the observed distribution of CV values between the 1st and 99th percentiles. The knots were located at CV values of 1.630%, 4.080%, 6.450%, and 11.617%. The *p* values were 0.016 for the overall association and 0.101 for nonlinearity. Duan’s smearing factor was treated as fixed when calculating the confidence intervals.

For CV, potential nonlinearity was assessed using a restricted cubic spline with four knots. The knots were placed at 1.630%, 4.080%, 6.450%, and 11.617%, corresponding to the 5th, 35th, 65th, and 95th percentiles in the extended complete-case sample of 3917 participants. Robust Wald tests were used to evaluate the overall association and the nonlinear component. Figure 2 presents sample-average marginal predictions of arithmetic mean triglyceride concentrations. A rug plot shows the distribution of CV.

Missingness was summarized for the outcome and for each covariate. The 3917 participants included in the extended complete-case model were compared with excluded participants using standardized mean differences (SMDs). Among all 5203 participants with observed triglyceride concentrations, missing covariate values were multiply imputed using 50 bootstrap imputations with predictive mean matching. Estimates were combined using Rubin’s rules.

Inverse probability of inclusion weighting (IPW) was also evaluated. Because the weights were unstable, analyses were conducted using both untruncated weights and weights truncated at the first and 99th percentiles. All analyses were performed using R version 4.5.1. A two-sided *p* value < 0.05 was considered statistically significant.

## 3. Results

### 3.1. Participant Characteristics

Of the 9500 eligible adults, 5203 had triglyceride measurements in 2009. The core Model B analysis included 5191 participants, and 3917 participants had complete data for the extended socioeconomic model. Characteristics of the extended complete-case sample according to CV quartile are presented in Table 1.

Among the 9500 eligible participants, 4297 did not have triglyceride measurements. By construction, CV, ASV, baseline age, sex, weight slope, and number of weight measurements had no missing values. Among the 5203 participants with observed triglyceride concentrations, data were missing for working status in 12 participants, education in 24, individual income in 1281, baseline BMI in six, smoking in 14, and alcohol use in 13 (Table S1).

The 3917 extended complete cases differed from excluded participants on several measured characteristics. For example, the absolute standardized mean difference (SMD) for the number of weight measurements was 0.457 when complete cases were compared with all excluded eligible participants and 0.628 when they were compared with participants missing triglyceride measurements. The corresponding absolute SMDs for working status were 0.872 when complete cases were compared with all excluded participants and 1.310 when they were compared with participants who had triglyceride measurements but incomplete extended-model covariate data (Table S2). These imbalances suggest the possibility of selection bias.

### 3.2. Weight Variability and Triglycerides

In the core sample, each 1 SD increment in CV was associated with 2.7% higher triglyceride concentrations, whereas each 5 kg increment in ASV was associated with 10.7% higher triglyceride concentrations (Table 2).

Within the BMI-complete sample of 5185 participants, the reference estimates without baseline BMI were 2.6% (95% CI, 0.7–4.6%) per 1 SD increment in CV and 10.7% (95% CI, 5.9–15.8%) per 5 kg increment in ASV. After further adjustment for baseline BMI in the same sample, the corresponding estimates were 3.6% (95% CI, 1.7–5.6%) and 5.6% (95% CI, 1.0–10.3%), respectively. Compared with the same-sample reference models, the log-scale CV coefficient increased by 36.9%, whereas the ASV coefficient was attenuated by 46.9%. Neither finding should be interpreted as demonstrating independence from adiposity.

In the extended socioeconomic model of 3917 participants, each 1 SD increment in CV was associated with 2.9% higher triglyceride concentrations (95% CI, 0.5–5.4%), and each 5 kg increment in ASV was associated with 12.2% higher triglyceride concentrations (95% CI, 6.2–18.6%).

### 3.3. Shape of the Association Between CV and Triglycerides

The four-knot restricted cubic spline showed an overall association between CV and triglyceride concentrations (*p* for overall association = 0.016; Figure 2). The test for nonlinearity was not statistically significant (*p* for nonlinearity = 0.101).

Predicted triglyceride concentrations generally increased across the observed range of CV. However, the data did not provide strong evidence that the nonlinear model fit better than a model containing only a linear CV term. This finding should not be interpreted as evidence of strict linearity, particularly because uncertainty increased in regions where observations were sparse.

### 3.4. Missing-Data and Other Sensitivity Analyses

In the multiply imputed extended socioeconomic model among all 5203 participants with observed triglyceride concentrations, the estimates were similar to those from the core complete-case analysis. Each 1 SD increment in CV was associated with 2.7% higher triglyceride concentrations (95% CI: 0.7% to 4.6%; *p* = 0.007), and each 5 kg increment in ASV was associated with 10.5% higher triglyceride concentrations (95% CI: 5.7% to 15.5%; *p* < 0.001; Table S3).

In the 3917-person extended complete-case sample, the truncated IPW estimates were 4.2% for CV (95% CI: 1.1% to 7.3%) and 11.1% for ASV (95% CI: 3.5% to 19.3%; Table S3). The untruncated weights were highly right-skewed, and truncation increased the effective sample size from 672 to 2410; therefore, the IPW findings should be interpreted cautiously (Table S6).

After 2009 smoking and alcohol use were added to the extended model, the estimates were 3.1% for CV (95% CI: 0.7% to 5.5%; n = 3915) and 12.3% for ASV (95% CI: 6.2% to 18.7%; Table S3). Occupational physical-activity components were available for 2263 complete cases, all of whom were current workers. In this selected subgroup, the association for CV was null and imprecisely estimated, whereas that for ASV remained positive (Table S3). Confidence intervals based on community-clustered standard errors also preserved the direction of the core estimates (Table S3).

Dietary data were available for 3867 of the 3917 participants in the extended socioeconomic model (98.7%). In this diet-complete sample, the same-sample reference model without dietary adjustment estimated 2.8% higher triglyceride concentrations per 1 SD increment in CV (95% CI: 0.4% to 5.2%; *p* = 0.022) and 11.9% higher concentrations per 5 kg increment in ASV (95% CI: 5.8% to 18.2%; *p* < 0.001). After adjustment for log total energy intake and fat energy percentage, the corresponding estimates were 2.8% (95% CI: 0.4% to 5.3%; *p* = 0.021) and 11.9% (95% CI: 5.9% to 18.3%; *p* < 0.001), respectively. Estimates were also essentially unchanged in the energy-only model, when carbohydrate energy percentage was used instead of fat energy percentage, and in the extreme-intake sensitivity analysis excluding participants with energy intake below the first or above the 99th percentile (Table S3).

Overall, these sensitivity analyses did not eliminate the possibility of residual confounding by lifestyle factors. In particular, smoking, alcohol use, physical activity, and dietary intake were measured concurrently with triglycerides; therefore, the corresponding models should be interpreted as contemporaneous sensitivity analyses rather than as control of temporally established baseline confounding.

### 3.5. Absolute-Effect Estimates

Marginal standardization translated the extended socioeconomic model to the observed mmol/L scale. Increasing CV from a z score of 0 to 1 corresponded to an adjusted arithmetic mean triglyceride difference of 0.048 mmol/L (95% CI: 0.008 to 0.089), whereas moving from the 25th to the 75th percentile of CV corresponded to a difference of 0.059 mmol/L (Table S5). Increasing ASV from the sample median of 2.77 kg to 7.77 kg corresponded to a difference of 0.200 mmol/L (95% CI: 0.094 to 0.306), and the ASV 25th-to-75th percentile contrast corresponded to a difference of 0.086 mmol/L (Table S5).

The absolute differences were modest at typical exposure contrasts. In particular, a 5 kg ASV increment was large relative to the observed ASV distribution (median, 2.77 kg; IQR, 1.80–4.07 kg; Table 1). Statistical significance therefore should not be equated with individual clinical importance.

## 4. Discussion

In this CHNS analysis, greater long-term weight variability was associated with higher triglyceride concentrations measured in 2009. These associations remained after adjustment for directional weight change and the number of weight measurements. In the core model, each 1 SD increment in CV was associated with 2.7% higher triglyceride concentrations. Each 5 kg increment in ASV was associated with 10.7% higher concentrations. The estimates remained positive after multiple imputation and additional adjustment for socioeconomic factors.

Adjustment for baseline BMI affected the two variability measures differently. The CV coefficient increased by 36.9%, whereas the ASV coefficient decreased by 46.9%. This difference suggests that CV and ASV capture distinct features of long-term weight history. Their relationships with baseline body size may also differ.

The direction of our findings is consistent with previous studies that have linked greater weight variability to cardiovascular events and mortality [3,4,6,7,8,9,10,14,15]. Direct evidence for triglycerides and other intermediate lipid outcomes is more limited. Comparisons across studies are also difficult because the definitions of weight variability vary considerably. Previous studies have used the SD or CV of repeated weights, variability independent of the mean, successive weight changes, or predefined cycles of weight loss and regain. Study populations, follow-up periods, and adjustment strategies have also differed. These measures describe related aspects of weight history, but they are not interchangeable. Our analysis adds to this evidence by examining two complementary measures while accounting for each participant’s long-term weight slope.

CV and ASV describe different properties of longitudinal weight patterns. CV measures the overall dispersion of repeated weights relative to the mean body weight. ASV captures the average absolute difference between consecutive measurements [3,4]. Because ASV is expressed in kilograms, people with greater body weight may experience larger absolute changes even when their proportional changes are similar to those of lighter individuals. The reduction in the ASV coefficient after adjustment for baseline BMI suggests that part of the association may reflect differences in baseline body size.

The increase in the CV coefficient after BMI adjustment is more difficult to interpret. CV is a relative measure because mean body weight appears in its denominator. It therefore has a different mathematical relationship with BMI than ASV does. The coefficient increase may reflect the correlation structure among CV, BMI, and mean body weight. It may also reflect a suppression effect. These explanations remain tentative because the present analysis was not designed to determine why the coefficient changed after adjustment.

The findings should also be considered on the original triglyceride scale. Marginal standardization yielded an adjusted arithmetic mean difference of 0.048 mmol/L per 1 SD increment in CV and 0.200 mmol/L per 5 kg increment in ASV. The difference associated with ASV was larger, although its interpretation depends on the distribution of ASV in the study population. These estimates represent average between-participant differences rather than the triglyceride change expected after modifying an individual’s weight pattern. Triglyceride concentrations show substantial within-person variation, and a single measurement may not fully represent an individual’s usual long-term triglyceride status [16]. The absolute differences were modest at typical exposure contrasts and should not be interpreted as evidence of individual clinical benefit. This study was not designed to assess clinical events, treatment effects, clinical thresholds, risk reclassification, or incremental predictive value beyond established metabolic risk factors. Because established risk factors were not evaluated using directly comparable exposure scales and contrasts, direct quantitative comparisons with those factors are not warranted.

Several biological processes could contribute to the observed associations. Repeated changes in energy balance may affect lipid mobilization, lipid storage, hepatic triglyceride handling, and adipose-tissue function [17,18,19,20,21,22]. These mechanisms are most directly relevant to repeated changes in energy intake and adiposity, including intentional weight loss followed by regain. However, the variability measures used in this study were not specific to intentional weight cycling. Similar CV or ASV values could arise from illness-related weight loss, pregnancy-related changes, aging, differences in measurement timing, or natural long-term variation. The available CHNS data could not determine which of these processes accounted for the observed associations.

The restricted cubic spline analysis supported an overall association between CV and triglyceride concentrations. It did not provide strong evidence of nonlinearity. This result indicates that the nonlinear spline terms did not clearly add information beyond the linear component. It does not establish a strictly linear association across the entire CV range. Confidence intervals widened in areas with fewer observations, particularly at higher CV values. Estimates at the extremes of the distribution should therefore be interpreted cautiously.

Adjustment for participant-specific weight slope helped distinguish variability around the long-term trajectory from sustained weight gain or loss [23]. Adjustment for the number of measurements addressed differences in the opportunity to observe weight changes. However, neither adjustment fully accounted for differences in measurement timing.

ASV was calculated from the absolute differences between consecutive weight measurements and was not standardized for the interval between survey waves. A 5 kg change over a short interval therefore contributed as much to ASV as a 5 kg change over a longer interval. Previous studies using data collected at irregular visits have addressed this issue by dividing successive absolute differences by the corresponding time intervals [24,25]. Longer intervals may provide more opportunity for larger weight changes to occur. Adjustment for the number of measurements and long-term weight slope cannot fully account for this feature of the ASV calculation. This limitation should be considered when interpreting the ASV results.

The BMI sensitivity analysis showed that the ASV association was sensitive to baseline body size, whereas the CV association increased after adjustment. These estimates should not be interpreted as independent of adiposity. A single baseline-BMI measurement could not characterize adiposity throughout 1989–2006. BMI is also an indirect measure of adiposity and cannot distinguish the contributions of fat mass and fat-free mass to total body weight [26,27].

Working status, measured in 2009, was retained as a concurrent indicator of employment-related social and occupational circumstances rather than as a direct measure of physical activity. Because it was assessed after the period used to calculate weight variability and concurrently with triglycerides, it was not treated as a temporally established baseline confounder. Accordingly, its inclusion should be interpreted as concurrent covariate adjustment, and the findings should not be interpreted as independent of employment-related or socioeconomic conditions.

Residual confounding remains an important concern. Adjustment for current smoking and past-year alcohol use did not materially reduce the associations. However, both variables were measured in 2009 and may represent concurrent correlates rather than prior confounders. Occupational physical-activity data were available only for a selected subgroup of current workers. In this subgroup, the CV estimate was imprecise and close to the null, whereas the ASV estimate remained positive. The restricted sample limits the interpretation and generalizability of these results.

The lack of triglyceride measurements before or during the weight variability period is another important limitation. We could not determine whether greater weight variability preceded an increase in triglyceride concentrations. We also could not distinguish such an increase from persistently higher triglyceride levels. Underlying metabolic disease or other illness may have influenced both previous weight patterns and triglyceride concentrations in 2009. Measuring weight variability before the 2009 outcome reduces concerns about simultaneous measurement, but it does not eliminate reverse causation.

Selection should also be considered. More than 45% of eligible participants did not have a triglyceride measurement in 2009. Participants included in the extended complete-case model differed from excluded participants in the number of weight measurements, working status, age, and other observed characteristics.

Multiple imputation showed that the estimates were not highly sensitive to missing covariate data among participants with observed triglyceride measurements. It did not address missing outcome data. IPW estimates were in the same direction as the primary estimates, but extreme weights and low effective sample sizes reduced their stability. Selection bias may remain if the availability of triglyceride measurements was related to both previous weight patterns and triglyceride concentrations. The direction of any resulting bias cannot be determined from the available data.

Several limitations warrant cautious interpretation. This observational study could not distinguish intentional, illness-related, or other causes of weight change. Survey waves occurred at irregular intervals, triglycerides were measured only once, and outcome and socioeconomic data were substantially incomplete. Repeated lipid measurements, detailed body-composition data, and information on intentional weight change were unavailable. Several lifestyle factors were unavailable, measured concurrently with triglycerides, or available only in selected subgroups. Although the associations were materially unchanged after adjustment for contemporaneously measured 3-day energy intake and macronutrient composition, this short dietary assessment may not represent habitual diet and may itself have been influenced by preceding weight change, health status, or clinical advice. Residual confounding and reverse causation therefore cannot be excluded, and the dietary analyses should not be interpreted as control of baseline dietary confounding or as evidence of a causal association.

## 5. Conclusions

Greater long-term weight variability was associated with modestly higher subsequent triglyceride concentrations in Chinese adults. The association persisted in several sensitivity analyses, but baseline BMI materially attenuated the ASV estimate, and residual confounding and selection remain plausible. These findings support further study of weight trajectories with better information on intentionality, illness, diet, and physical activity; they do not establish causality, independence from obesity, or individual clinical benefit.

## Figures and Tables

**Table 1:** Characteristics of the extended socioeconomic complete-case sample by quartile of weight CV.

CV Quartile (Empirical Range, %)	Age (Years)	Men, n (%)	Baseline BMI (kg/m^2^)	Weight Slope (kg/Year)	Weight Measurements, n	Working in 2009, n (%)
Q1, n = 980 (0.00–3.40)	40.21 ± 12.23	516 (52.7)	22.27 ± 3.00	0.045 ± 0.223	4.60 ± 1.41	661 (67.4)
Q2, n = 979 (3.40–5.11)	38.73 ± 11.25	475 (48.5)	22.33 ± 2.84	0.133 ± 0.364	5.13 ± 1.41	694 (70.9)
Q3, n = 979 (5.11–7.60)	37.67 ± 10.95	456 (46.6)	21.98 ± 2.67	0.290 ± 0.486	5.25 ± 1.40	662 (67.6)
Q4, n = 979 (7.60–23.93)	34.46 ± 10.75	485 (49.5)	21.50 ± 2.66	0.588 ± 0.740	5.17 ± 1.36	743 (75.9)

Values are mean ± SD or n (%). CV ranges are empirical ranges in the 3917-person sample. The overall CV was 5.71 ± 3.13% (median 5.11%, IQR 3.40–7.60); the overall ASV was 3.21 ± 1.97 kg (median 2.77 kg, IQR 1.80–4.07). Descriptive differences are not presented as hypothesis tests.

**Table 2:** Associations of weight variability with triglyceride concentrations in primary and sensitivity models.

Model	Exposure	n	β (95% CI)	% Difference in (95% CI)	*p* Value	*p* Value
Core Model B	CV per 1 SD	5191	0.026 (0.007, 0.045)	2.7 (0.7, 4.6)	0.007	0.007
Core Model B	ASV per 5 kg	5191	0.102 (0.057, 0.147)	10.7 (5.9, 15.8)	<0.001	<0.001
Core Model B, BMI-complete reference	CV per 1 SD	5185	0.026 (0.007, 0.045)	2.6 (0.7, 4.6)	0.007	<0.001
Core Model B, BMI-complete reference	ASV per 5 kg	5185	0.102 (0.057, 0.147)	10.7 (5.9, 15.8)	<0.001	0.016
Core Model B + baseline BMI	CV per 1 SD	5185	0.036 (0.017, 0.054)	3.6 (1.7, 5.6)	<0.001	
Core Model B + baseline BMI	ASV per 5 kg	5185	0.054 (0.010, 0.098)	5.6 (1.0, 10.3)	0.016	
Extended SES model	CV per 1 SD	3917	0.029 (0.005, 0.052)	2.9 (0.5, 5.4)	0.016	0.016
Extended SES model	ASV per 5 kg	3917	0.115 (0.060, 0.170)	12.2 (6.2, 18.6)	<0.001	<0.001

Core Model B adjusted for baseline age, sex, weight slope, number of weight measurements, and working status in 2009. The BMI-complete reference and baseline-BMI models used the same sample (n = 5185); the latter additionally included baseline BMI. The extended socioeconomic model additionally included 2009 education and individual-income quintile. HC3 95% CIs are shown. Percent differences were calculated as [exp(β) − 1] × 100. Abbreviations: ASV, average successive variability; BMI, body mass index; CI, confidence interval; CV, coefficient of variation; SES, socioeconomic status.

## Data Availability

The data analyzed in this study are available to registered users of the China Health and Nutrition Survey, subject to the survey’s data-use terms. Analysis code and supporting outputs are available from the corresponding author on reasonable request, subject to CHNS requirements.

## Supplementary Materials

The following supporting information can be downloaded at: https://www.mdpi.com/article/10.3390/nu18162605/s1, Table S1: Missing data for the outcome and analysis variables; Table S2: Characteristics of extended-model complete cases and excluded participants, with standardized mean differences; Table S3: Missing-data, lifestyle, dietary, and cluster-robust sensitivity analyses; Table S4: Restricted cubic spline specification; Table S5: Model-based absolute triglyceride differences; Table S6: Inverse-probability-of-selection weighting diagnostics.
