# Supplementary material for: Long-Term Weight Variability Is Associated with Higher Triglyceride Concentrations After Accounting for Long-Term Weight Slope
Source: Nutrients. 2026 Aug 10;18(16):2605. doi: 10.3390/nu18162605 (PMC13516792; doi:10.3390/nu18162605)
Supplement: Supplementary file 1 [file nutrients-18-02605-s001.zip › nutrients-4450511-supplementary.pdf]

Long-term weight variability is associated with higher triglyceride concentrations after accounting for long-term weight slope

Miaomiao Xu 1, Huiguo Wang [2,3,*], Lin Zhu [2,3,*], Xiaoguang Liu [2,3,*]

[1] College of Physical Education, Guangdong University of Education, Guangzhou 510303, China; miaomiaoxu@gzucm.edu.cn
[2] College of Sports and Health, Guangzhou Sport University, Guangzhou 510500, China
[3] Research Center for Innovative Development of Sports and Healthcare Integration, Guangzhou Sport University, Guangzhou 510500, China
* Correspondence: 11450@gzsport.edu.cn (H.G.); 11251@gzsport.edu.cn (L.Z.); liuxg@gzsport.edu.cn (X.L.); Tel.: +86-020-38024021

Supplementary Tables

Table S1. Missing data for the outcome and analysis variables

Variable	Eligible participants (n=9,500): missing n (%)	TG observed (n=5,203): missing n (%)
Triglycerides	4,297 (45.2)	0 (0.0)
Weight CV	0 (0.0)	0 (0.0)
ASV	0 (0.0)	0 (0.0)
Baseline age	0 (0.0)	0 (0.0)
Sex	0 (0.0)	0 (0.0)
Weight slope	0 (0.0)	0 (0.0)
Number of weight measurements	0 (0.0)	0 (0.0)
Working status, 2009	3,645 (38.4)	12 (0.2)
Baseline BMI	13 (0.1)	6 (0.1)
Education level	3,658 (38.5)	24 (0.5)
Individual income	5,085 (53.5)	1,281 (24.6)
Three-day energy intake, 2009	3,713 (39.1)	76 (1.5)
Three-day carbohydrate intake, 2009	3,713 (39.1)	76 (1.5)
Three-day fat intake, 2009	3,713 (39.1)	76 (1.5)
Three-day protein intake, 2009	3,713 (39.1)	76 (1.5)
Current smoking, 2009	3,648 (38.4)	14 (0.3)
Past-year alcohol use, 2009	3,647 (38.4)	13 (0.2)
Occupational activity components, 2009	6,743 (71.0)	2,780 (53.4)

CV, coefficient of variation; ASV, average successive variability; BMI, body mass index; TG, triglycerides. Exposure variables and core demographic/trajectory variables were complete by construction in the eligible analytic file. Three-day dietary variables were derived from the 2009 CHNS constructed dietary-value file (c12diet).

Table S2. Characteristics of extended-model complete cases and excluded participants, with standardized mean differences

Characteristic	Included (n=3,917)	All excluded	SMD	Missing TG	SMD	TG observed, missing extended covariate	SMD
Baseline age, years	37.77 (n=3,917)	40.94 (n=5,583)	−0.231	40.52 (n=4,297)	−0.196	42.33 (n=1,286)	−0.363
Men, %	49.3 (n=3,917)	47.0 (n=5,583)	0.047	50.5 (n=4,297)	−0.023	35.3 (n=1,286)	0.287
Baseline BMI, kg/m²	22.02 (n=3,912)	21.89 (n=5,575)	0.043	21.84 (n=4,290)	0.062	22.08 (n=1,285)	−0.021
Weight slope, kg/year	0.264 (n=3,917)	0.250 (n=5,583)	0.023	0.257 (n=4,297)	0.011	0.226 (n=1,286)	0.071
Weight measurements, n	5.04 (n=3,917)	4.41 (n=5,583)	0.457	4.19 (n=4,297)	0.628	5.11 (n=1,286)	−0.052
Working in 2009, %	70.5 (n=3,917)	30.5 (n=1,938)	0.872	58.0 (n=664)	0.263	16.2 (n=1,274)	1.310
Weight CV, z score	−0.041 (n=3,917)	0.029 (n=5,583)	−0.071	0.018 (n=4,297)	−0.060	0.065 (n=1,286)	−0.110
ASV, 5-kg units	0.642 (n=3,917)	0.700 (n=5,583)	−0.134	0.706 (n=4,297)	−0.148	0.678 (n=1,286)	−0.087

Values are means or proportions calculated among participants with observed data for each characteristic. Standardized mean differences (SMDs) were calculated as the extended-model complete-case group minus the corresponding comparison group; therefore, negative values indicate lower values in the included group. Absolute SMDs ≥0.10 were considered indicative of potentially meaningful imbalance. Weight CV z scores were standardized using the mean and SD among all 9,500 eligible participants. ASV was expressed in 5-kg units. Baseline BMI was not required for inclusion in the extended socioeconomic complete-case sample, resulting in five participants with missing baseline BMI in the included group. Working status was measured in 2009 and was observed for only 1,938 of all excluded participants, including 664 participants with missing TG and 1,274 participants with observed TG but missing at least one extended-model covariate; SMDs for working status should therefore be interpreted cautiously.

Abbreviations: ASV, average successive variability; BMI, body mass index; CV, coefficient of variation; SD, standard deviation; SMD, standardized mean difference; TG, triglycerides.

Table S3. Missing-data, lifestyle, dietary, and cluster-robust sensitivity analyses

Analysis	Exposure	n	β (95% CI)	% difference (95% CI)	P value
50-imputation pooled model	CV per 1 SD	5,203	0.026 (0.007, 0.045)	2.7 (0.7, 4.6)	0.007
50-imputation pooled model	ASV per 5 kg	5,203	0.100 (0.055, 0.144)	10.5 (5.7, 15.5)	<0.001
Core TG-observation IPW, truncated	CV per 1 SD	5,191	0.035 (0.006, 0.064)	3.5 (0.6, 6.6)	0.019
Core TG-observation IPW, truncated	ASV per 5 kg	5,191	0.096 (0.024, 0.169)	10.1 (2.5, 18.4)	0.009
Extended complete-case IPW, truncated	CV per 1 SD	3,917	0.041 (0.011, 0.071)	4.2 (1.1, 7.3)	0.007
Extended complete-case IPW, truncated	ASV per 5 kg	3,917	0.105 (0.034, 0.176)	11.1 (3.5, 19.3)	0.004
Extended + 2009 smoking/alcohol	CV per 1 SD	3,915	0.030 (0.007, 0.054)	3.1 (0.7, 5.5)	0.012
Extended + 2009 smoking/alcohol	ASV per 5 kg	3,915	0.116 (0.060, 0.171)	12.3 (6.2, 18.7)	<0.001
Employed PA-sample reference	CV per 1 SD	2,263	0.004 (−0.028, 0.037)	0.4 (−2.8, 3.7)	0.796
Employed PA-sample reference	ASV per 5 kg	2,263	0.094 (0.019, 0.168)	9.8 (1.9, 18.3)	0.014
Employed + occupational physical activity	CV per 1 SD	2,263	0.004 (−0.029, 0.036)	0.4 (−2.8, 3.7)	0.818
Employed + occupational physical activity	ASV per 5 kg	2,263	0.093 (0.018, 0.167)	9.7 (1.8, 18.2)	0.015
Core, community-clustered SE	CV per 1 SD	5,191	0.026 (0.005, 0.048)	2.7 (0.5, 4.9)	0.016
Core, community-clustered SE	ASV per 5 kg	5,191	0.102 (0.054, 0.150)	10.7 (5.6, 16.1)	<0.001

All models used natural log-transformed TG as the outcome. Percentage differences were calculated as $[\exp(\beta) - 1] \times 100$. The multiply imputed extended socioeconomic model used 50 bootstrap predictive-mean-matching imputations, and estimates were combined using Rubin's rules with HC3 within-imputation covariance matrices. IPW models used stabilized inverse-probability-of-selection weights truncated at the 1st and 99th percentiles. Smoking, alcohol use, and occupational physical activity were measured in 2009 and were examined as exploratory sensitivity covariates. The occupational physical-activity analysis was restricted to 2,263 participants with complete component data, all of whom were current workers. The same-sample reference model included the extended-model covariates except working status, which had no variation; the adjusted model additionally included total occupational physical-activity minutes, divided by 1,000 for numerical scaling. Community-clustered models included 208 communities.

Abbreviations: ASV, average successive variability; CI, confidence interval; CV, coefficient of variation; HC3, heteroskedasticity-consistent covariance estimator; IPW, inverse-probability-of-selection weighting; PA, physical activity; SD, standard deviation; SE, standard error; SES, socioeconomic status; TG, triglycerides.

Table S3. (continued)

Analysis	Exposure	n	β (95% CI)	% difference (95% CI)	P value
Extended SES, diet-complete reference	CV per 1 SD	3,867	0.028 (0.004, 0.051)	2.8 (0.4, 5.2)	0.022
Extended SES, diet-complete reference	ASV per 5 kg	3,867	0.112 (0.057, 0.167)	11.9 (5.8, 18.2)	<0.001
Extended SES + log total energy	CV per 1 SD	3,867	0.028 (0.004, 0.051)	2.8 (0.4, 5.3)	0.021
Extended SES + log total energy	ASV per 5 kg	3,867	0.113 (0.057, 0.168)	11.9 (5.9, 18.3)	<0.001
Extended SES + log energy + fat energy percentage	CV per 1 SD	3,867	0.028 (0.004, 0.051)	2.8 (0.4, 5.3)	0.021
Extended SES + log energy + fat energy percentage	ASV per 5 kg	3,867	0.113 (0.057, 0.168)	11.9 (5.9, 18.3)	<0.001
Extended SES + log energy + carbohydrate energy percentage	CV per 1 SD	3,867	0.028 (0.004, 0.051)	2.8 (0.4, 5.3)	0.020
Extended SES + log energy + carbohydrate energy percentage	ASV per 5 kg	3,867	0.113 (0.058, 0.169)	12.0 (6.0, 18.4)	<0.001
Energy P1–P99 sensitivity: log energy + fat energy percentage	CV per 1 SD	3,789	0.025 (0.001, 0.049)	2.5 (0.1, 5.0)	0.038
Energy P1–P99 sensitivity: log energy + fat energy percentage	ASV per 5 kg	3,789	0.109 (0.053, 0.164)	11.5 (5.4, 17.9)	<0.001

Dietary sensitivity analyses used three-day average dietary variables from the 2009 CHNS constructed dietary-value file (c12diet). Dietary intake and TG were measured concurrently; therefore, dietary variables were evaluated as sensitivity covariates rather than temporally established baseline confounders. Total energy intake was log-transformed. Fat and carbohydrate energy percentages were calculated as 100 × fat (g) × 9 / total energy (kcal) and 100 × carbohydrate (g) × 4 / total energy (kcal), respectively, and were examined in separate models. Diet-complete reference models were refitted in the identical sample used for dietary adjustment.

Table S4. Restricted cubic spline specification

Parameter	Value
Analysis sample	Extended socioeconomic complete cases (n=3,917)
Number of knots	4
Knot percentiles	5th, 35th, 65th, 95th
Knot locations (CV, %)	1.630, 4.080, 6.450, 11.617
P overall (HC3)	0.0159
P for non-linearity (HC3)	0.1005
Duan smearing factor	1.2260
Displayed CV range	0.886% to 14.834%

The spline adjusted for baseline age, sex, weight slope, number of weight measurements, 2009 working status, education, and individual-income quintile. Overall and non-linearity tests used HC3 covariance.

Table S5. Model-based absolute triglyceride differences

Exposure contrast	Predicted TG at lower exposure value (mmol/L)	Predicted TG at higher exposure value (mmol/L)	Adjusted difference, higher − lower (95% CI), mmol/L
CV: z = 0 (5.850%) to z = 1 (9.236%)	1.657	1.706	0.048 (0.008, 0.089)
CV: 25th percentile (3.40%) to 75th percentile (7.60%)	1.623	1.682	0.059 (0.011, 0.107)
ASV: median (2.77 kg) to median + 5 kg (7.77 kg)	1.636	1.835	0.200 (0.094, 0.306)
ASV: 25th percentile (1.80 kg) to 75th percentile (4.07 kg)	1.600	1.686	0.086 (0.046, 0.128)

Predictions are sample-average marginal arithmetic-mean triglyceride concentrations from separate extended socioeconomic linear models for CV and ASV, retransformed to mmol/L using Duan’s smearing method. CV z scores were standardized using the mean (5.850%) and SD (3.386 percentage points) among all 9,500 eligible participants. Percentiles and the ASV median were calculated in the extended complete-case sample (n = 3,917). Confidence intervals were obtained from 2,000 parametric simulations of the regression coefficients using the HC3 covariance matrix; the model-specific Duan smearing factors were treated as fixed.

Table S6. Inverse-probability-of-selection weighting diagnostics

Selection target	Selected/eligible, n	Selection-model AUC	Untruncated stabilized weight, median (P1–P99; maximum)	Untruncated ESS	Truncated weight range; ESS
Observed 2009 TG	5,203/9,500	0.793	0.757 (0.669–8.703; 90.302)	416	0.669–8.703; 2,628
Extended complete case	3,917/9,500	0.745	0.734 (0.599–6.318; 51.703)	672	0.599–6.318; 2,410

IPW, inverse-probability weighting; AUC, area under the receiver operating characteristic curve; ESS, effective sample size. Stabilized selection weights among selected participants were truncated at the 1st and 99th percentiles. ESS was calculated as $(\Sigma w)^2/\Sigma w^2$, where w denotes the stabilized selection weight. The large maximum weights and low untruncated ESS indicate substantial weight instability in both selection models; therefore, the IPW estimates should be interpreted cautiously.
